# Decreasing utilization and increasing prices of brand-name oral contraceptive pills: Implications to societal costs and market competition

**DOI:** 10.1371/journal.pone.0234463

**Published:** 2020-06-11

**Authors:** James X. Zhang

**Affiliations:** Department of Medicine, The University of Chicago, Chicago, IL, United States of America; University of Jyvaskyla, FINLAND

## Abstract

The affordability of pharmaceuticals has been a major challenge in US health care. Generic substitution has been proposed as an important tool to reduce the costs, yet little is known how the prices of more expensive brand-name drugs would be affected by an increased utilization of generics. We aimed to examine the trend of overall utilization and the total costs of brand-name oral contraceptive pills (OCPs), the most widely used form of contraception, and its association with the pharmaceutical market concentration among the OCPs. Data from the Medical Expenditure Panel Survey (MEPS) 2011–2014, a nationally representative survey of healthcare utilization, were extracted on the utilization of generic and brand-name OCPs. A multiple logit regression analysis was conducted to assess the trend in utilization of brand-name OCPs over time. Total costs, including the costs to the payers and consumers, were synthesized. The Herfindahl-Hirschman Index (HHI), an index describing market concentration, was constructed, and a multiple regression analysis was conducted to evaluate the association between the brand-name OCP prices and the market share of individual brand-name drugs. The odds of utilizing brand-name drugs decreased steadily in 2012, 2013, and 2014 compared to 2012 (AOR 0.87, 0.73, 0.55, respectively, p<0.05) controlling for patient mix. Despite significant decline in total utilization, there was a 90% increase in the price of brand-name OCPs, resulting an 18% increase in revenue from 2011 to 2014 for the industry. During this time, pharmaceutical market concentration for OCPs increased (HHI increased from 1105 in 2011 to 2415 in 2014). Each percentage point increase in the market share by a brand-name OCPs was associated with a $3.12 increase in its price. Market mechanisms matter. Practitioners and policy makers need to take market mechanisms into account in order to realize the benefits of generic substitutions.

## Introduction

Rising pharmaceutical prices are a persistent and serious challenge in the US healthcare system [1]. Thirty-five million American adults (19%) between 19- and 64-years-old did not fill a prescription due to cost in 2014 [2]. Generic substitution, for which the government or insurers seek to reduce costs by stimulating the prescription and dispensing of generally cheaper generic medicines instead of their more expensive branded equivalents drugs, has been a popular tool for drug cost containment in the US and beyond [3, 4]. The central promise of generic substitution is that, because generic drug applicants for drug approval by the government do not have to repeat animal and clinical (human) studies that were required of the brand-name medicines to demonstrate safety and effectiveness, the costs of producing generic drugs should be lower. The hope is that this will be translated into lower costs to the consumers and society and that the utilization of branded equivalent drugs will be reduced. It is unclear, however, if the brand-name drug prices decrease when faced with increasing utilization of generic drugs in order to be competitive. More importantly, even with a reduction in utilization of brand-name drugs, society may not receive the benefit of a reduction of total costs, if the brand-name drugs increase their prices in the market. These are important practice and policy questions because, without a clear answer, the benefits of generic substitution will be in question.

This study focused on the utilization and price trend of the oral contraceptive pills (OCPs), the most widely used form of contraception [5, 6]. We aimed to test whether the overall price of brand-name OCPs can increase in a concentrated market, even while there is increasing utilization of generic OCPs and decreasing utilization of brand-name OCPs. We expect the larger brand-name manufacturers will be able to weather the storm of generic competition by leveraging their market positions to raise prices.

As a result, the overall costs of brand-name OCPs to the society may increase despite the decrease in utilization. We think the answers to these research questions may have far-reaching implications for medical practice, health policy, drug manufacturing, and the efficiency of the US healthcare system [7].

## Methods

We used the Medical Expenditure Panel Study (MEPS) 2011–2014 dataset to examine the trend in utilization and prices in brand-name OCPs. The MEPS is a large-scale, nationally representative survey of families and individuals, their medical providers, and employers across the United States, and it is the most complete source of data on the cost and use of health care and health insurance coverage [8]. The MEPS has a detailed Prescribed Medicine file, including drug brand names, quantity, days of supply, and total payments (both out-of-pocket and from public and private insurers), linked with a Full-year Consolidated Data file for respondents’ demographics, insurance coverage, and survey weights. In this study, we extracted both generic and brand-name drugs in utilization measured by prescriptions filled and a key drug price metric to the society: the total price including out-of-pocket payments (OOPs) and all payments from all payers, both public and private, including Medicare, Medicaid and TriCare. The total payments from all payers reflect the overall cost of the commodity in the market to society. We refer to this as the “price” to society. The brand-name OCPs were identified through the RXNAME field which identifies the drug names in the Prescribed Medicine file and the field of FDA approval, i.e., New Drug Application (NDA) and Abbreviated New Drug Application (ANDA) for the generic drugs [9]. We standardized the price per prescription for OCPs to a 28-day supply (the most common prescription length) and adjusted the price to value in 2014 dollars using the Bureau of Labor Statistics medical price index [10].

### Trend in utilization of brand-name OCPs

We analyzed the trend in utilization of brand-name OCPs by developing a multiple logit regression model, comparing the odds of utilizing a brand-name OCP vs. a generic OCP across the years from 2011 to 2014. During this time period, the economy was in steady growth after the deep recession that began 2008, and there was no major political shock or anticipation of change of federal government administration before a tumultuous US election in 2015. Thus, this time period provides a clear background for observing the market forces in the drug sector. This analysis was conducted at the prescription level because we are interested in the likelihood that a brand-name OCP is filled, while controlling for all other factors. The dependent variable in the multiple logit model was a binary indicator of filling a brand-name OCP Rx, and the independent variables were the years of Rx with 2011 as the referent and 2012, 2013, and 2014 as dummy indicators, respectively, and other covariates of patient characteristics, including age, race, ethnicity, insurance status (Medicare, Medicaid, private insurance, or no insurance coverage). We included these variables to control the patient mix so that the odds of utilizing brand-name OCPs can be compared across years.

### Market concentration

One potential explanation of the large firms’ ability to raise prices for brand-name OCPs when faced with strong generic competition is, based on classical economic theory, that large firms in more concentrated markets, i.e., with fewer competitors, will have the ability to raise price because of their larger market power [11]. This analysis is complicated by the fact that pharmaceutical markets do not abide by the assumptions of classical economists’ model of competition, since there are two distinct types of products in the market: brand-name and generics. While generics are highly competitive in terms of prices, there is a cumulative body of literature indicating that a significant proportion of physician, pharmacists, and lay people perceive generics as inferior to the brand-name drugs in terms of quality, safety, and side effects [12, 13, 14]. Hence, the large brand-name manufacturers could take advantage of such perception and leverage their large market share to increase prices despite increasing utilization of generics. Women who are using OCPs on a continuous basis might be particularly receptive to such a marketing strategy, since the perception of quality and side-effect is critical. We constructed the Herfindahl-Hirschman Index (HHI), a commonly accepted measure of market concentration [15], to describe the concentration in sales revenue volume in the brand-name OCP market. The HHI is estimated using the market share of each brand manufacturer’s sale revenues. This estimation incorporates price, since a company’s ability to charge a high price even with moderate sales volume is a common result of heightened market power. To further ascertain the association between the market share of an individual brand and its prices, we conducted a multiple regression analysis to regress the individual brand’s market share on its prices, controlling for the year, and patients case mix including age, race, ethnicity, and insurance status.

### Three-dimensional analysis of price, quantity sold, and market share

To visualize the complex relationship among price, quantity sold, and market share, we conducted a three-dimensional analysis to contrast the market in 2011 and 2014. The purpose of this exercise is to illustrate how 1) in a less concentrated, more competitive market, there is a general downward sloping of demand-price relationship, meaning the manufacturers would not be able to raise prices while increasing their sales volume; 2) however, in a more concentrated market, there could be an upward-sloping relationship between the individual manufacture’s’ prices and sale volume because of the market power that the larger brand-name manufacturers can wield, despite the overall decreasing utilization of brand-name drugs.

### Sample exclusions and standardization

For all analyses, we only included women between ages 18 and 55 years for both OCPs and reference cases. This would have excluded those women who used OCPs for the purpose of other than contraception [16], which reduces the heterogeneity among the patient mix. All analyses were weighted using the person’s sample weight in the MEPS to reflect the national market estimates.

### Ethics statement

There were no human subjects in this study. An approval from the Institutional Review Board is hence not required. There is no funding for this study.

## Results

**Fig 1:**
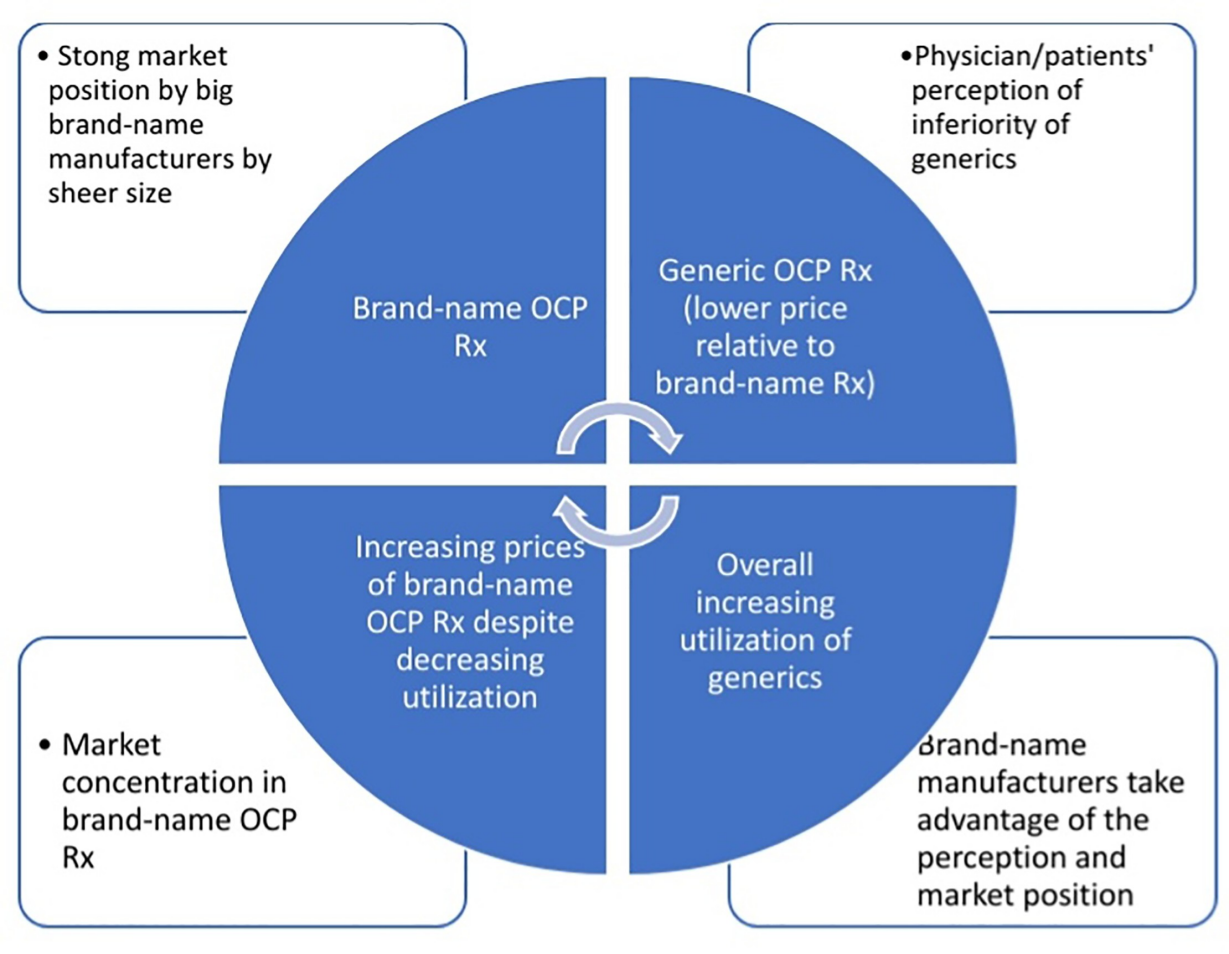
Conceptual framework of market competition between brand-name and generic OCP drugs.

The conceptual framework for this work is illustrated in Fig 1. We theorized that brand-name manufacturers take advantage of the perception of inferior quality of generics and their own superior position in the concentrated drug market to raise their prices, despite strong price competition from and increasing utilization of generic drugs.

Table 1 shows the patient characteristics including age, race, ethnicity, and insurance status with and without weighing for the year of 2011, 2012, 2013, and 2014, respectively. Altogether, there were 4,562, 4,331, 3,930, and 3,677 unique observations of OCP prescriptions filled in each year by 867, 556, 551, and 529 women aged between 18 and 55, respectively, representing 10,723,684, 6,254,298, 6,415,649, and 6,034,767 American women, respectively. The weighted average numbers of OCP Rx filled per person per year were 5.6 (s.d. 4.1), 5.2 (s.d. 3.6), 5.2 (s.d. 3.7), 5.2 (s.d. 3.8), respectively, with total days of supply per person per year 234 (s.d. 192), 222 (s.d. 166), 210 (s.d. 171). and 212 (s.d. 170), respectively.

**Table 1:** Patient characteristics from 2011 to 2014.

	2011 (unweighted/ weighted)	2012 (unweighted/ weighted)	2013 (unweighted/ weighted)	2014 (unweighted/ weighted)
N	867/10,723,684	556/6,254,298	551/6,415,649	529/6,034,767
Age: Mean (s.d.)	31(9)/31(9)	30 (9)/30(9)	30(9)/30(9)	29(9)/29(9)
African American: N (%)	113(13) / 759,558 (7)	72 (13)/ 446,269(7)	91(17)/ 566,307 (9)	73(14)/ 400,033 (7)
Other race: N (%)	79 (9)/ 706,530(7)	54 (10)/ 463,093(7)	52(9)/ 321,932(5)	67(13)/ 586,073(10)
Hispanic: N (%)	145(17)/ 1,015,055 (9)	120 (22)/ 652,079(10)	118 (21)/ 655,793(10)	140(26)/ 806,333(13)
Medicare: N (%)	8(1)/91,792(1)	8(1)/67,333(1)	5(1)/53,246(1)	5(1)/44,304(1)
Medicaid: N (%)	495(11)/ 4,688,590(8)	546(13)/ 4,560,550(8)	736(19)/ 5,827,156(12)	686(19)/ 5,292,499(11)
Tricare: N (%)	69(2)/ 6,052,456(1)	65(2)/ 597,573(1)	67(2)/ 801,686(2)	24(1)/ 211,347(0)
Private Insurance: N (%)	3,737(82)/ 51,712,025 (85)	3,506(81)/ 45,903,988(84)	3,055(78)/ 4,2971,807(86)	2,881(78)/ 42,358,542(87)
No insurance: N (%)	349(8)/ 4,255,500(7)	362 (8)/ 5,111,095(9)	277(7)/ 2,198,184(4)	204(6)/ 1,570,871(3)
Number of OCP Rx Filled PPPY: N(s.d.)	5.3(3.9)/5.6(4.1)	4.9(3.6)/5.2(3.6)	4.9(3.7)/5.2(3.7)	4.8(3.7)/5.2(3.8)
Total Days of Supply PPPY: N (s.d.)	214(178)/ 234(192)	205(165)/ 222(166)	197(170)/ 210(171)	195(165)/ 212(170)

Insurance status are not mutually exclusive as patients could have multiple insurance coverage at the same time, hence the total percentage do not add to 1. PPPY: per person per year.

From the multiple logit model, the odds ratios of filling up an brand-name OCPs vs. generic OCPs in 2012, 2013, and 2014 compared to 2011 declined steadily over time from 0.91 in 2012, to 0.67 in 2013, and 0.57 in 2014 (p<0.01, respectively), controlling for age, race, ethnicity, and insurance status (Table 2). The tight 95% confidence intervals were driven in part by the weighting to reflect the national trend.

**Table 2:** Odds ratio of filling a brand-name OCP prescription relative to generic OCP prescription controlling for socio-demographic variables and insurance status 2011–2014.

Variable of Interest	Odds Ratio	95% Confidence Interval
Year		
2011	referent	referent
2012	0.91	0.91–0.91
2013	0.67	0.67–0.67
2014	0.52	0.52–0.52
Age	0.99	0.99–0.99
Race		
White	referent	referent
African American	0.92	0.93–0.94
Other race	0.93	0.84–1.07
Ethnicity		
Non-Hispanic	referent	referent
Hispanic	0.99	0.99–0.99
Insurance coverage		
Private insurance	referent	referent
Medicare	1.32	1.32–1.32
Medicaid	1.09	1.08–1.09
Tricare	1.36	1.35–1.36
No insurance coverage	1.27	1.27–1.27

The outcome variable is the utilization of brand-name OCPs (binary variable with 1 indicating brand-name OCPs, and 0 generic OCPs) for the years of 2011, 2012, 2013, and 2014 using MEPS. The analysis was weighted.

**Fig 2:**
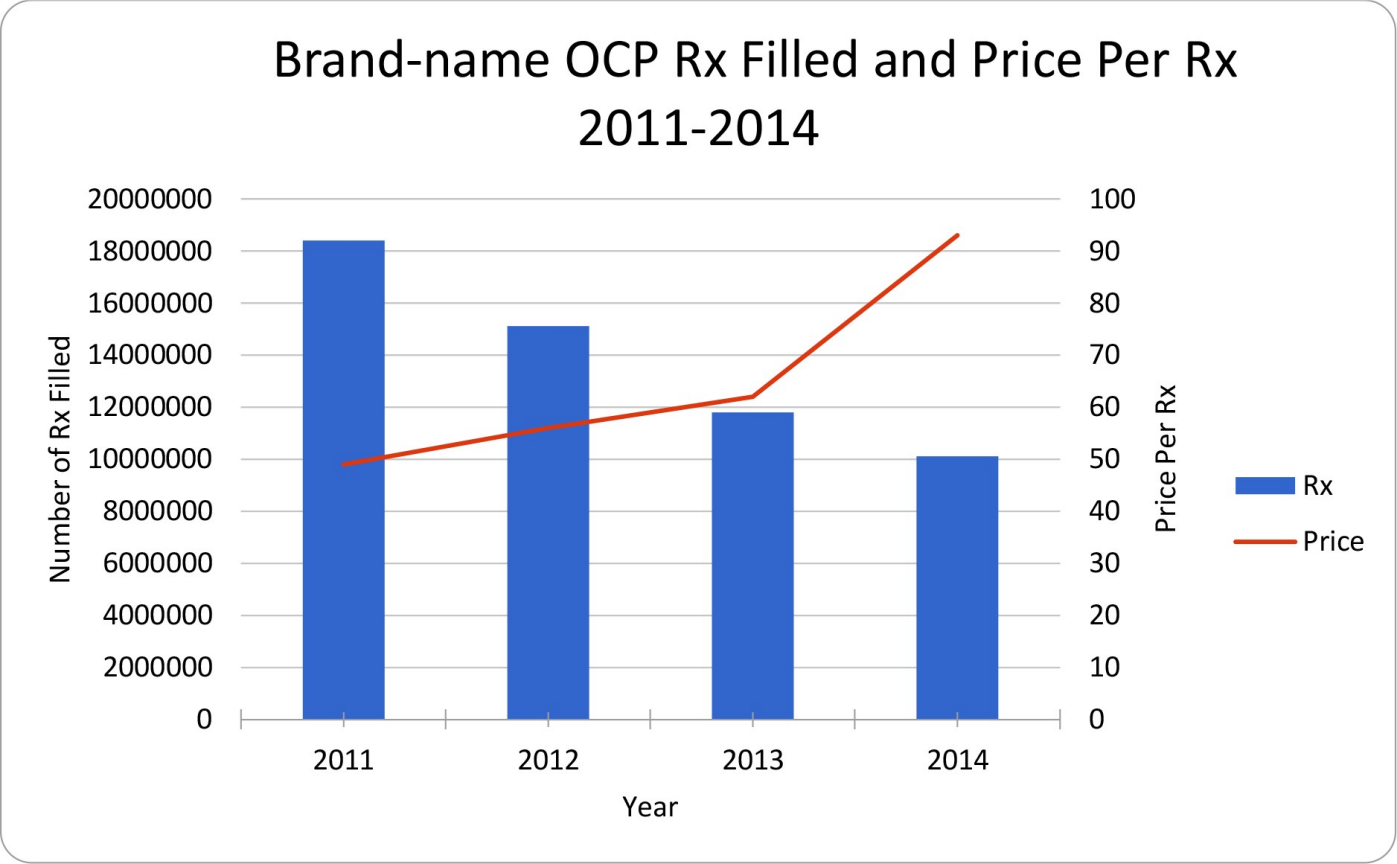
Brand-name OCP Rx filled and price per Rx 2011–2014. OCP: oral contraceptive pills. The results are based on MEPS. Prices per Rx are the summation of all payments from all payers. The price per Rx was quantity-adjusted to 28-day supply and price-index-adjusted to the 2014 dollar.

**Fig 3:**
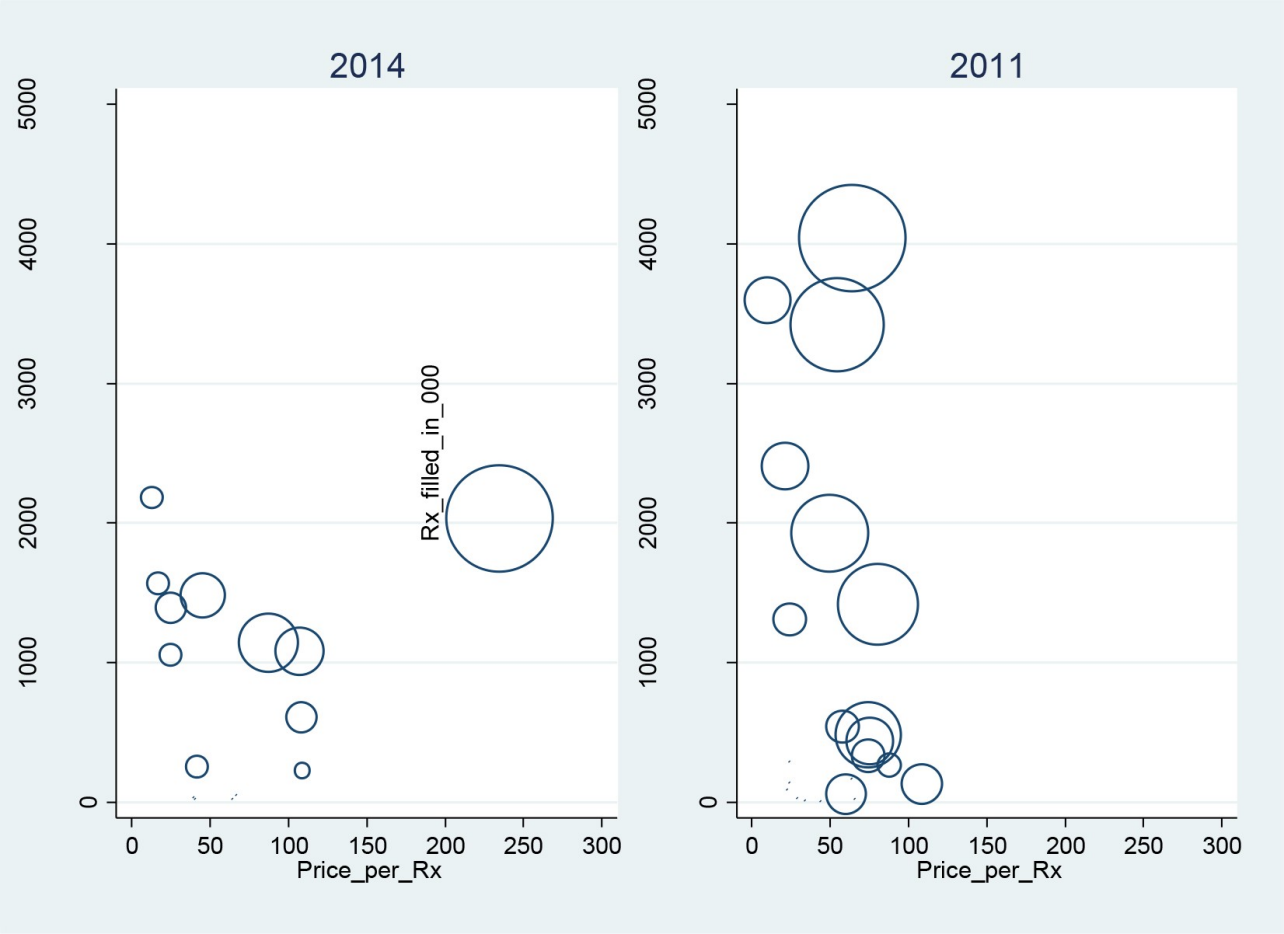
A three-dimensional relationship among prescription prices, prescriptions filled, and market share by individual brand-name manufactures, 2014 and 2011. Prices are the summation of all payments from all payers. The price and quantity per brand was quantity-adjusted to 28-day supply and price-index-adjusted to the 2014 dollar, and weighted to reflect the market. The size of the circles represents the market share of each brand.

While the total volume of brand-name OCP prescriptions filled declined steadily over time, the average prices of brand-name OCP increased by 90% from 2011 to 2014 (Fig 2). In 2011, market shares by each brand were more evenly distributed with prices of each brand spans narrowly differentiated; in contrast, in 2014, market shares by each brand were much more differentiated with an upward relationship between the price-prescription filled for those manufacturers with larger market shares. There was also a reduction of total number of brands from 22 in 2011 to 16 in 2014 (Fig 3). There was a steady increase of HHI from 1105 in 2011 to 2415 in 2014, and despite 37% fewer brand-name OCP prescriptions filled, there was an 18% increase in revenue from 2011 to 2014 for the industry (Table 3). The multiple regression analysis showed that for every 1 percentage point increase in the market share by an individual brand, its price increased by $3.12 (p<0.01) controlling calendar years, age, race, ethnicity, and insurance status. For the covariates, on average, compared to whites, African American had a price $4.78 lower (p<0.01) per Rx; compared to non-Hispanics, Hispanics had a price $4.87 higher per Rx; compared to those with private insurance, those with Medicare, uninsured had a price $7.02, $5.72 lower per Rx (p<0.01 respectively), while those with Medicaid, Tricare $1.26, $15.83 higher per Rx (p<0.01 respectively); each year of increase in age was associated with $0.32 higher in price (p<0.01); each year of increase in calendar year was associated with $7.57 higher per Rx (p<0.01).

**Table 3:** Total sales revenue, prescriptions filled, and Herfindahl-Hirschman index in the brand-name oral contraceptive market from 2011 to 2014.

	2011	2012	2013	2014
Total Brand-name Prescriptions Filled (1000s)	18,423	15,095	11,841	10,096
Total Revenue (US$ Millions)	1,201	1,075	946	1,312
Herfindahl-Hirschman Index	1,105	1,637	1,333	2,451

Revenues were price-index-adjusted to the 2014 dollar and weighted to reflect the market.

## Discussion and conclusions

In this study, we have shown that with the increasing utilization of generic OCPs, the overall costs of brand-name OCPs did not decrease; on the contrary, the total costs to the society actually increased despite a sharp drop in total number of brand-name OCPs filled. The coexistence of increased total costs and reduced utilization was largely driven by increased prices of the brand-name OCPs, accompanied by the increasingly concentrated OCP market. This may have important implications for medical practitioners, health policy makers, and the pharmaceutical industry.

First, generic substitution alone may not necessarily reduce the total costs to the society. The central promise of generic substitution is that the increased utilization of less expensive generics would lead to the lower total costs. However, with the market mechanism favoring the large brand-name manufacturers in their ability to raise prices, even with decreased utilization, the total costs of the brand-name drugs could still increase, resulting in the loss of welfare to the consumers and increased profits to the manufacturers.

Second, market concentration may play an important role in firm’s ability to raise price, because larger firms will be able to charge a higher price without fear of being undercut by the smaller competitors [11]. The United States Department of Justice generally classifies markets into three types: unconcentrated markets (HHI below 1500); moderately concentrated markets (HHI between 1500 and 2500), and highly concentrated markets (HHI above 2500), when considering the cases for potential consequences of industry mergers and acquisition [17]. However, these guidelines have not often been used in relation to drug markets. More research is greatly needed to understand how the pharmaceutical markets function to aid policymakers in lowering overall costs and improving drug access for society at large.

Third, it is noteworthy that the price increases in the brand-name OCPs were achieved under conditions of substantial competition from generics and increasing utilization of these generics over time. Our finding that generic competition does not necessarily lower brand-name drug prices is consistent with a recent brief that stated that inter-brand and brand versus generic competition failed to lower prices for consumers [18]. Our study suggested that such a perceived market failure could be actually a manifestation of a market mechanism that favors the large firms’ ability to raise price when the market is concentrated.

Such an increased market concentration with increased costs may have far-reaching implications for consumers and practitioners. Higher concentration means fewer choices for consumers, higher costs to society, and higher revenues to the top sellers. Higher profits for brand-name manufacturers could reinforce their incentive for direct marketing to increase market share and further distort the market, increasing the costs to society.

This study has implications for future policy. For example, given the nature of market competition in brand-name OCPs and the difference between brand-name and generic drugs, policy could be reformulated to gauge the relative prices of brand-name drugs to the generics, since these relative prices represent the true opportunity costs to society. This will provide a gauge on the loss of welfare due to increased prices of brand-name drugs and allow policy to be directed at improving the efficiency of the drug market to encourage competition.

Our study has several limitations. First, we were not able to identify the insurance plans of patients, and we did not control for geographic variations. It is possible that prices vary by plans and regions, so further investigations are required to capture such nuanced variations. Second, the MEPS Prescription Medication file does not identify any rebates for medications. There is no reliable measure to estimate the rebates, and not accounting for these rebates might lead to a false underestimation of the drug costs borne by the entire health system [19]. Third, there could be other factors reinforcing the relationship between the market share and prices. The investigation of such factors is beyond the scope of this study. Future research should be directed at the rigorous analysis of a comprehensive set of factors influencing the complex fabric of relationship among generic drugs, brand-name drugs, prices, sale volume, market share, and market concentration. Fourth, such a relationship between decreasing utilization and increasing prices in other brand-name pharmaceutical products is worth further investigation, but it is beyond the scope of this study. Past studies have suggested that physicians’ and nurse practitioners’ perception of generics may vary across different types of pharmaceuticals, as do the perceptions of lay people [20, 21]. Our study might indicate that manufacturers could take advantage of such perception of inferior quality of generics to maximize their profits at the expense of society at large. There is a paucity of literature on how perception of generic drugs influences big pharmaceutical companies’ marketing strategies. More research is much needed to affirm the trade-off between the societal costs and manufacturers’ profits resulting from such strategies. Fifth, while research has shown that most drug classes had a generic substitution rate higher than 90% and men were more likely to utilize generics [22], the longer-term trend of generic substitution in the OCPs needs to be further assessed, and the impact of gender on such a trend to be evaluated.

In summary, despite substantial decreasing utilization of brand-name OCPs, the total price to society increased. The market mechanism matters, and the concentration in the brand-name OCP market may have played a role in facilitating such price increases. Practitioners and policy makers need to take the market mechanism into account in order to realize the benefits of generic substitutions.

## Supporting information

S1 Data (DTA)
